# 

**Published:** 1892-07-09

**Authors:** 


**Notices of Births, Deaths, and Marriages,** are inserted in this column at a charge of 2s. 6d. for an announcement not exceeding four lines.

### NOTICE TO CORRESPONDENTS.

All MS., Letters, Books for Review, and other matters intended for the Editor should be addressed THE EDITOR, THE LODGE, PORCHESTER SQUARE, LONDON, W.

The Editor cannot undertake to return rejected M.S., even when accompanied by stamped directed envelope.

**Notice to Advertisers and Subscribers.**

All Advertisements, Orders for Copies of THE HOSPITAL, and Business Communications should be addressed to The Publisher, *not to the Editor*, THE HOSPITAL, 140, Strand, W.C.

The Cost of Subscription, post free, is as follows:—Per week, 2½d.; per quarter, 2s. 6d.; per half-year, 5s.; per annum, 8s. 6d. Foreign:—Per annum, 10s. 8d.; payable, in all cases, in advance.

**"Burdett's Hospital Annual," 1891—1892.**

Third year of publication. 600 pp. Boards, gilt lettering. A most complete guide to British and Colonial Hospitals, Dispensaries, Nursing and Convalescent Institutions, and Asylums. Price 3s. 6d. Published by THE SCIENTIFIC PRESS (Limited), 140, Strand, W.C.

**NOTICE.—The Index for Vol. XI. (ending March, 1892) is on sale at the Office of this Journal, price 2½d., post free.**

### Scraps and Gleanings.

On Hospital Sunday the collections at Aldborough Church, Boroughbridge, amounted to £19 10s. 8¼d.

The amount of subscriptions announced at the festival of the Royal Masonic Institution for Boys amounted to £12,212.

Sir Savile Crossley, M.P., has repeated this year his generous gift of £1,000 towards the Hospital Sunday Fund.

The North-West London Hospital has benefited to the extent of £270 by the "Old English Market Fair," held in the Camden Road.

The benefit matinée at the Criterion, in aid of the East London Hospital for Children, has been postponed to Tuesday, the 12th inst.

The Duke of Westminster has contributed £500 to the Chester Infirmary, an amount realised by one shilling admissions to Eaton Hall.

The Earl of Dunraven has accepted the office of President of St. Peter's Hospital, in place of the late Lieutenant-General Lord Abinger.

The twentieth annual report of the Lytham Cottage Hospital, just issued, states that 90 patients were admitted, or 26 more than the previous year.

The Marquess of Dufferin, as the Chancellor of Dublin University, will present the congratulatory address at the Trinity College Tercentenary, to be held at Dublin.

The Press Association reports that Sir William Aitken, Professor of Pathology, at the Army Medical School, Netley, has died at Southampton. He was 67 years of age.

The new wing to the Leeds General Infirmary, which has been erected at a cost of nearly £40,000, was opened last week by Sir Andrew Fairbairn, the High Sheriff of Yorkshire.

Dr. A. P. Luff, Lecturer on Medical Jurisprudence, and Physician to St. Mary's Hospital, has been appointed Official Analyst to the Home Office, in the place of the late Dr. Meymott Tidy.

Colonel Campbell, retiring House Governor of the Royal National Hospital for Consumption, Ventnor, has received a handsome travelling clock and illuminated address by the inmates of the Institution.

Princess Christian, who was accompanied by Prince Christian and Princess Victoria of Schleswig-Holstein, presented the prizes on Thursday last to the students of the Royal Holloway College, Egham.

Lady Courterneere has bequeathed £28,000 towards the Gibbing's Memorial Wing of the Cork Charitable Infirmary. The wing is to be three storeys high, and will be fitted up with every necessary and modern appliances.

Mr. W. Hughes, Secretary of the Cancer Hospital, Brompton, has been presented with a testimonal consisting of a telescopic brass and copper lamp by the Matron, nurses, and resident Medical Officers, as a token of their esteem.

An interesting little musical service was held in the chapel of St Mary's Hospital, Paddington, last week, when Mr. Richards, organist of Christ Church, Lancaster Gate, gave an organ recital, which was much enjoyed by patients, nurses, and visitors.

The joint hospital for infectious diseases for the districts of Brighouse, Rastrick, Clifton, and Hartshead was opened early last month. The hospital is erected on a site at Thornhills, Clifton, which has been obtained on a seven years' lease, with option of purchase.

The new buildings recently added to the Salisbury Infirmary were opened by the Bishop of the Diocese. The ceremony took place in the new accident ward which was prettily decorated with palms, flowers, and ferns. A sum of £1,500 is required to pay off the debt.

Dr. Milton, of the Kasr Elain Hospital, Cairo, has recovered one thousand pounds damages for libel in an action brought by him against the French journal *Bosphore*, which published a violent attack on the English doctors and surgeons connected with that institution.

Lord Sandhurst presided at the annual festival dinner in connection with the Metropolitan Hospital. During the evening subscriptions and donations to the amount of £2,472 were announced, which included £1,000 for the endowment of a cot to the memory of Mr. John Carter.

Thanks to the kindness and generosity of Mr. Ruston, the old out-patients' department of the Lincoln Hospital has been converted into a ward for children, and an entirely new pavilion for out-patients, with drug stores, board-room, &c., has been built at a cost of £4,000.

Miss J. C. Flemming, late of St. John's Wood, has left £2,000 to the General Fund, and £1,000 to the Building Fund of the Cheyne Hospital for Sick and Incurable Children, £500 each to the Royal Free Hospital (Gray's Inn Road) and University College Hospital, besides legacies to various other charities.

Mr. Dampier Palmer has presented to the Gravesend Hospital a cup to be competed for annually by the football teams of the Borough of Gravesend, Northfleet, and Perry Street. At each tie or match gate-money is to be charged, and the proceeds handed over to the Hospital. The cup is to be called the Gravesend Hospital Charity Cup.

The Metropolitan Hospital Sunday Fund now amounts to nearly £35,000, including £1,208 from St. Michael's, Chester Square; £1,002, St. Jude's, South Kensington; a further sum of £1,000 from Sir Savile Crossley, M.P.; £440, All Saints', Ennismore Gardens; and £363, St. Mary Abbott's Parish Church, Kensington Mission Hall, and open-air collection.

A chemist's assistant at Northampton has been sentenced to 10 months' imprisonment for selling pills to various druggists at 2s. a box, stating he was travelling for a patent medicine firm. Instead of this being the case, the pills were purchased from a Birmingham firm for 2d. a box. We are not told whether the pills have been analysed, but happily poisons are generally dear.

During the past year 24 entirely new nurses and servants' bedrooms (now occupied) have been added to the Wigan Infirmary. The laundry has been rebuilt and fitted with all necessary modern appliances. The drainage system throughout the hospital has been reconstructed. The operating theatre and adjoining wards have been altered and enlarged, and an entertainment room for patients has been fitted up under the North Ward.

### THE STUDENT OF MEDICINE.

[All communications for this Supplement should be addressed to THE EDITOR of THE HOSPITAL, at 140, Strand, with the words, "Students' Column," written in the left hand top corner of the envelope.]

### APPOINTMENTS.

Arnison, W. C., M.D.Durh., M.R.C.S., appointed Professor of Surgery to the University of Durham. Bradshaw, T. R., M.D., M.R.C.P.Lond., M.R.C.S., appointed Assistant Honorary Physician to the Royal Infirmary, Liverpool. Bristowe, Hubert C., M.D.Lond., appointed Medical Officer at the Bath and Somerset Asylum. Browning, G. Dansey, M.R.C.S., L.R.C.P. Lond., appointed Resident Obstetric Assistant to Westminster Hospital. Bulman, F., M.B., C.M.Durh., appointed Medical Officer to the Workhouse, Newport, Mon. Byles, J. B., M.R.C.S., L.R.C.P.Lond., appointed Senior House Physician to Westminster Hospital. Carling, W., M.B., B.C.Cantab., appointed Assistant House Surgeon to Guy's Hospital. Devereux, W. C., M.A., M.B., B.C.Cantab., M.R.C.S., L.R.C.P., appointed Acting Honorary Surgeon to the Tewkesbury Hospital. Durham, H. E., M.B., B.C.Cantab., appointed House Surgeon to Guy's Hospital. Hamilton, George G., M.B., C.M.Edin., appointed Assistant Honorary Surgeon to the Royal Infirmary, Liverpool. Havelock, J. G., M.B., C.M.Edin., reappointed Senior Assistant Medical Officer to the Royal Asylum, Montrose. Howden, J. C., M.D.Edin., re-appointed Medical Superintendent to the Royal Asylum, Montrose. Jewell, J. W. F., M.B.Lond., appointed Assistant House Physician to Guy's Hospital. Lewis, H. W., M.R.C.S., L.R.C.P.Lond., appointed Senior House Surgeon to the Westminster Hospital. Luff, Arthur P., M.D.Lond., Lecturer on Medical Jurisprudence, and Physician to St. Mary's Hospital, appointed Official Analyst to the Home Office. Mann, J. Dixon, F.R.C.P., appointed Professor of Medical Jurisprudence at Owens College, Manchester. Mills, H. H., M.R.C.S., L.R.C.P. Lond., appointed Junior House Physician to Westminster Hospital. Nelson, Thomas, M.D.Edin., appointed Ingleby Lecturer at Queen's College, Birmingham. Rake, A. T., M.B., B.S.Lond., appointed House Surgeon to Guy's Hospital. Riddell, J. Scott, M.B., C.M., M.A.Aber., appointed Medical Officer in Charge of Oldmacher Poorhouse. Salter, C. E., M.B., B.S.Lond., appointed House Physician to Guy's Hospital. Soutar, R., M.B., C.M.Aber., re-appointed Medical Officer to the Royal Infirmary, Montrose. Stone, V., M.D.St.And., F.R.C.S. Edin., reappointed Medical Officer to Royal Infirmary, Montrose. Tebbs, W. H. Alisonn, L.R.C.P.Lond., M.R.C.S., late House Surgeon to Westminster Hospital, appointed House Surgeon to Addenbrooke's Hospital, Cambridge. Thomas, A., M.B., B.S. Lond., appointed House Physician to Guy's Hospital. Thomas, W. Thelwall, L.R.C.P.Edin., F.R.C.S.Eng., appointed Assistant Honorary Surgeon to the Royal Infirmary, Liverpool. Wingrave, V. H. Wyatt, M.R.C.S.E., L.S.A., late Registrar and Anæsthetist, appointed Assistant Surgeon to the Central London Throat and Ear Hospital. Young, F. C., M.B., B.C.Cantab., appointed Assistant House Physician to Guy's Hospital.

### VACANCIES.

The following vacancies are announced this week, the amount of salary in each case being given after the name of the institution:

**Resident Medical Officers.**

Ancoats Hospital, Manchester, Junior Resident Surgeon—Salary, £50 per annum, with board, &c.
Dundee Royal Lunatic Asylum, Assistant Medical Officer—Salary, £100 per annum, with board, &c.
East London Hospital for Children, Glamis Road, Shadwell, E., House Surgeon—Board, &c.
East London Hospital for Children, Glamis Road, Shadwell, E., House Physician—Board, &c.
Royal Albert Hospital, Devonport, Assistant House Surgeon—Board, &c.
Royal South Hants Infirmary, Southampton, Assistant House Surgeon—Board, &c.
Royal United Hospital, Bath, House Surgeon; must be M.R.C.S.—Salary, £60 per annum, with board, &c.
Wirral Children's Hospital, Woodchurch Road, Birkenhead, Resident House Surgeon, lady or gentleman—Salary, £50 per annum, with board, &c.
Wolverhampton Eye Infirmary, House Surgeon—Salary, £60 per annum, with board, &c.

**Non-Resident Medical Officers.**

Dental Hospital of London, Leicester Square, W.C., Assistant Dental Surgeon; must be L.D.S.
McGill University, Montreal, Canada, Professor of Pathology for the Faculties of Medicine and Comparative Medicine—Salary, £400 per annum.
St. Thomas's Hospital Medical School, Obstetric Tutor—Salary, £50 per annum.

### THE STUDENT OF MEDICINE—(*continued*).

### NOTES FROM THE SCHOOLS.

The Charing Cross Hospital Medical School.—At the invitation of the medical officers and lecturers of the Charing Cross Hospital Medical School, a large company assembled at the school in Chandos Street, Charing Cross, yesterday afternoon, when Mr. J. Passmore Edwards distributed the prizes to the students. Mr. S. Boyd, the Dean, read the annual report, which commenced by stating that the extension of the premises begun in 1889 had been completed, and that the school building would now compare favourably with any of the kind in London. The academical year had been one of unusually hard work for the management, for the five years' curriculum became an actual fact with the beginning of the year, and many changes in and additions to the school curriculum had been necessary to adapt it to the requirements of the conjoint board. Biology, insanity, public health, practical midwifery, and ophthalmology had become compulsory subjects. For the teaching of biology, the school had secured the help of Mr. Chalmers Mitchell, while psychological medicine would be undertaken by Dr. Hack Tuke. In regard to the students, there had been 84 new entries during the year, made up of 45 general students, 28 dental students, and 11 occasional students. There had been an average daily attendance of about 240, and the standard of work was surely rising. The Llewellyn Scholarship, certificate, and £25 were handed to Mr. G. H. Hooper; Mr. D. P. Gabell won the Golding Scholarship and £15; Mr. S. L. J. Steggall the Governors' clinical gold medal; and Mr. E. Brown the Pereira prize. Other chief prizes distributed were to Mr. W. C. D. Hills and Mr. F. H. Atkinson (anatomy), Mr. G. B. Clarke, Mr. D. C. Rees, and Mr. D. P. Gabell (physiology), Mr. J. R. Langley and Mr. D. P. Gabell (chemistry), Mr. G. L. Austen (osteology), Mr. N. R. J. Rainier (medicine), Mr. W. T. White (practical medicine), Mr. W. J. Robertson (surgery), Mr. V. P. Foote (therapeutics), Mr. G. B. Clarke (materia medica), Mr. P. J. Probyn (midwifery), Mr. W. T. White (forensic medicine and toxicology), Mr. H. H. Phillips (pathology), Mr. H. H. Woods (public health), and Mr. A. J. Stevens (dental surgery). At the conclusion of the presentation Sir Joseph Fayrer proposed a vote of thanks to Mr. Passmore Edwards for presiding, and the resolution was carried by acclamation.

### UNIVERSITY INTELLIGENCE.

#### Cambridge.

Degrees.—At the congregation on June 16th the following medical degrees were conferred:—

M.D.—F. R. B. Bisshopp, M.A., King's; H. Head, M.A., Trinity; C. G. May, M.A., Trinity; F. P. Weber, M.A., Trinity; R. N. Goodman, M.A., St. John's; J. M. Clarke, M.A., Caius; J. G. Adami, M.A., Jesus.

M.B. and B.C.—G. A. Coulby, F. J. Dixon, R. L. Langdon-Down, W. T. Lister, E. H. G. Morris, C. N. Thomas, W. W. Walker, all of Trinity; A. Carling, A. R. Cowell, H. S. Ware, W. S. West, F. C. Young, all of St. John's; G. T. Birdwood, Peterhouse; W. E. Drake and W. J. Horne, both of Clare; C. Buttar, Pembroke; G. H. A. C. Berkeley, G. P. Chappel, R. B. Ferguson, R. Freer, H. Troutbeck, all of Caius; H. L. Brooksbank, Trinity Hall; H. B. Bolus and J. W. Dixon, both of Jesus; W. Wood, Christ's; G. Wilkinson, sen., and F. N. Windsor, both of Emanuel; R. J. Reece, J. D. Thomas, C. C. Vigurs, all of Downing.

M.B.—L. Cobbett, Trinity.

From the *Ordo senioritatis* promulgated on June 21st it appears that during the academical year just closed, 22 graduates were admitted to the M.D., 74 to the M.B., and 69 to the B.C. degree. The numbers last year were 19, 72, and 70 respectively.

Course of Practical Instruction in Hygiene.—Dr. Anningson and Mr. Robinson, M.A, give notice that they will give a course of practical instruction in hygiene, commencing July 9th. The course will consist principally of practical instruction in the sanitary examination of water, air, and foods, chemically and microscopically, having special regard to the requirements of medical officers of health. It will also include the preparation of different media used in the cultivation of micro-organisms, and their cultivation therein by inoculation from air and water. Short lectures will be given in explanation of the methods employed, and of analytical reports in general relating to water, air, and foods, and the interpretations to be put on them. Short lectures will be given in the principles of ventilation, drainage, water supply, &c., illustrated by the aid of diagrams, models, &c., also on the legal and physical remedies to conditions injurious to health, and on statistical methods.

Demonstrator of Anatomy.—Mr. Edward Barclay-Smith, M.A., M.B., of Downing College, has been appointed a Junior Demonstrator of Anatomy, in succession to Mr. Michell, of Caius College.

